# Human Telomerase Reverse Transcriptase as a Therapeutic Target of Dihydroartemisinin for Esophageal Squamous Cancer

**DOI:** 10.3389/fphar.2021.769787

**Published:** 2021-10-22

**Authors:** Qingrong Li, Qiang Ma, Lei Xu, Chuanli Gao, Lihua Yao, Jilin Wen, Miyuan Yang, Jibing Cheng, Xi Zhou, Jiang Zou, Xiaowu Zhong, Xiaolan Guo

**Affiliations:** ^1^ Department of Clinical Laboratory, Affiliated Hospital of North Sichuan Medical College, Nanchong, China; ^2^ Translational Medicine Research Center, North Sichuan Medical College, Nanchong, China; ^3^ Department of Laboratory Medicine, North Sichuan Medical College, Nanchong, China

**Keywords:** dihydroartemisinin, esophageal squamous cancer, reactive oxygen species, telomerase reverse transcriptase, specificity protein 1

## Abstract

**Objective:** To elucidate the oncogenic role of human telomerase reverse transcriptase (hTERT) in esophageal squamous cancer and unravel the therapeutic role and molecular mechanism of dihydroartemisinin (DHA) by targeting hTERT.

**Methods:** The expression of hTERT in esophageal squamous cancer and the patients prognosis were analyzed by bioinformatic analysis from TCGA database, and further validated with esophageal squamous cancer tissues in our cohort. The Cell Counting Kit-8 (CCK8) and colony formation assay were used to evaluate the proliferation of esophageal squamous cancer cell lines (Eca109, KYSE150, and TE1) after hTERT overexpression or treated with indicated concentrations of DHA. Transwell migration assay and scratch assay were employed to determine the migration abilities of cancer cells. Fluorescence microscopy and flow cytometry were conducted to measure the intracellular reactive oxygen species (ROS) levels in cancer cells after treated with DHA. Moreover, RT-PCR and Western blot were performed to test the alteration of associated genes on mRNA and protein level in DHA treated esophageal squamous cancer cell lines, respectively. Furthermore, tumor-bearing nude mice were employed to evaluate the anticancer effect of DHA *in vivo*.

**Results:** We found that hTERT was significantly upregulated in esophageal squamous cancer both from TCGA database and our cohort also. Overexpression of hTERT evidently promoted the proliferation and migration of esophageal squamous cancer cells *in vitro*. Moreover, DHA could significantly inhibit the proliferation and migration of esophageal cancer cell lines Eca109, KYSE150, and TE1 *in vitro*, and significantly down-regulate the expression of hTERT on both mRNA and protein level in a time- and dose-dependent manner as well. Further studies showed that DHA could induce intracellular ROS production in esophageal cancer cells and down-regulate SP1 expression, a transcription factor that bound to the promoter region of hTERT gene. Moreover, overexpression of SP1 evidently promoted the proliferation and migration of Eca109 and TE1 cells. Intriguingly, rescue experiments showed that inhibiting ROS by NAC alleviated the downregulation of SP1 and hTERT in cells treated with DHA. Furthermore, overexpression of SP1 or hTERT could attenuate the inhibition effect of DHA on the proliferation and migration of Eca109 cells. In tumor-bearing nude mice model, DHA significantly inhibited the growth of esophageal squamous cancer xenografts, and downregulated the expression of SP1 and hTERT protein, while no side effects were observed from heart, kidney, liver, and lung tissues by HE stain.

**Conclusion:** hTERT plays an oncogenic role in esophageal squamous cancer and might be a therapeutic target of DHA through regulating ROS/SP1 pathway.

## Introduction

Esophageal cancer is a highly aggressive and lethal malignancy, which ranks the eighth in incidence and the sixth in mortality in the world according to world health statistics in 2018 (Bray et al., 2018). Esophageal squamous cell carcinoma (ESCC) and esophageal adenocarcinoma (EAC) are the most common histological subtypes of esophageal cancer. In China, esophageal squamous cell carcinoma accounting for about 90% of the esophageal cancers each year (Abnet et al., 2018). Risk factors of esophageal squamous cell carcinoma including tobacco, betel quid, alcohol beverages, pickled vegetables, hot foods, X-and γ-radiation, achalasia, and Fanconi anemia (Abnet et al., 2018). At present, the therapeutic strategies of esophageal cancer are including surgery, radiotherapy, chemotherapy, biological-targeted drug therapy, and combination therapy (Zhao et al., 2019). However, the prognosis of patients with esophageal cancer remains poor, with a 5-year survival rate of approximately 30–40% (Ferlay et al., 2015). The most important reasons were the complex pathogenesis of esophageal cancer (Napier et al., 2014).

Telomeres are “cap” structures located at the end of chromosomes and consist of repetitive “5′-TTAGGG-3′” DNA sequences, which maintain the integrity of chromosomes and the stability of genome. Telomeric DNA repeats are synthesized by a reverse transcriptase named telomerase. Telomerase consists of two subunits—the RNA subunit “human telomerase RNA” (hTR) and the protein subunit “human telomerase reverse transcriptase” (hTERT) (Blackburn, 2000). The hTERT mRNA expression is strictly controlled and closely associated with telomerase activity, which indicates that hTERT is the primary determinant of enzyme activity (Leao et al., 2018). Telomerase activity is usually barely detected in most normal somatic tissues except in germ cells and some stem cells (Ramlee et al., 2016). However, telomerase activity is highly elevated in cancer cells, due to the fact that most cancer cells achieve proliferation immortality by activating or up-regulating normally silenced hTERT (Shay, 2016). In recent years, hTERT promoter mutations and epigenetic alterations have been the focus of research. Reports have shown that hTERT promoter mutations are present in thyroid cancer and glioblastoma, and are associated with the expression of hTERT and elevation of telomerase activity (Vinagre et al., 2013). For patients with meningioma, in addition to high Ki-67, the presence of hTERT mutations may promote the tumor more aggressive and recurrent (Sahin et al., 2021). Moreover, hTERT genetic and epigenetic alterations are associated with hTERT upregulation (Seynnaeve et al., 2017), while only few scientists focus the effect of hTERT dysregulation on tumors.

Dihydroartemisinin (DHA), which is a semi-synthetic derivative of artemisinin, has been widely used as a first-line antimalarial treatment. In recent years, studies have shown that DHA can exert anti-tumor effects mainly by inducing cell cycle arrest (Lin et al., 2016), inducing ferroptosis (Yuan et al., 2020), inducing apoptosis (Liu et al., 2018), inhibiting tumor angiogenesis (Dong et al., 2014), inhibiting invasion and migration (Guo et al., 2021), etc., which showed that DHA has strong anticancer activities with different mechanisms. DHA inhibited the proliferation of ESCC *in vitro* and *in vivo* through the AKT1-mTOR-p70S6K signaling axis (Zhu et al., 2020). Our previous study also showed that DHA could inhibit the proliferation of esophageal cancer cells by inducing autophagy (Ma et al., 2020). DHA can also act as a sensitizer, inducing the activation of NF-κB by inactivating PDT, which enhanced PDT induced growth inhibition and apoptosis in esophageal cancer cells (Li et al., 2014). In addition, DHA was found to enhance the sensitivity of ESCC cells to cisplatin by attenuating the activation of Shh pathway (Cui et al., 2020). However, no study has yet reported the effect of DHA on hTERT expression in esophageal cancer.

Here, we aimed to explore the expression of hTERT in esophageal cancer and its oncogenic role on esophageal cancer cells, as well as the regulatory effect of DHA on the expression of hTERT in esophageal cancer cells and reveal the underlying mechanisms, providing a new theoretical basis for DHA as an anti-esophageal cancer drug in the future.

## Materials and Methods

### Reagents and Antibodies

DMEM and FBS were purchased from Gibco (Grand Island, United States). Dihydroartemisinin (DHA) was obtained from Must Biotechnology (Chengdu, China). Actinomycin D was purchased from Selleck Biotechnology (Houston, United States). NAC reagent, and ROS or CCK8 detection kits were purchased from Beyotime Biotechnology (Shanghai, China). pcDNA3.1–3×HA-TERT and pCMV-myc-SP1 plasmid were purchased from Hedgehobio (Shanghai, China). Lipofectamine 2000 reagent was provided by Invitrogen (Carlsbad, United States). The antibodies against SP1, Ki-67, and GAPDH were purchased from Cell Signaling Technology (Beverly, United States). The antibody against hTERT was purchased from Abcam (Cambridge, United Kingdom). Goat anti-Rabbit IgG was purchased from BOSTER (Wuhan, China).

### Cell Culture

Human esophageal squamous cancer cell lines Eca109, KYSE150, and TE1 were obtained from the Translational Medicine Research Center of North Sichuan Medical College and were cultured in DMEM medium containing 10% fetal bovine serum at 37°C in an atmosphere containing 5% CO_2_.

### Cell Viability Measurement

Human esophageal cancer cell lines Eca109, KYSE150, and TE1 were seeded into a 96-well plate at 8×10^3^ cells per well in DMEM containing 10% FBS. After 12 h, the cells were treated with DMSO or different concentrations of DHA for 48 h, or DHA at 100 μM for different time points, respectively. After DHA treatment, 10 μl of CCK8 reagent was added to each well and incubated for 2 h at 37°C in an atmosphere containing 5% CO_2_. The absorbance (A) was measured at 450 nm by a microplate reader (Sunrise™ microplate reader, TECAN, NC). All determinations were carried out in triplicate.

### Plate Colony Formation Assay

Esophageal cancer cells Eca109, KYSE150, and TE1 were seeded into 6-well plates at a density of 2×10^3^ cells per well, respectively. After 5 days, the medium was replaced with 2 ml of 10% FBS contained DMEM with DMSO or 100 μM DHA. The culture was terminated when clones were visible by naked eyes. Then cells were fixed with methanol for 10 min and stained with crystal violet staining solution for 10 min. Images were obtained using a GT-S650 scanner (Epson).

### Transwell Assay

Esophageal squamous cancer cells in logarithmic growth phase were seeded into 6-well plates at a density of 5 × 10^5^ per well and treated with DMSO or 100 μM DHA after 24 h. Forty-8 h later, cells were resuspended in serum-free medium by digestion with 0.25% trypsin and adjusted to a cell density of 5 × 10^5^/ml. A total of 200 μl cell suspension was added to upper chambers and 500 μl DMEM supplemented with 10% FBS was added to the lower chamber. After incubating for 24 h, the upper chamber was removed, fixed with methanol and stained with crystal violet solution, subsequently photographed under a microscope.

### Scratch Assay

Esophageal cancer cell lines were seeded into 6-well plates at a density of 5 × 10^5^ per well. The cell layer was scratched with a 10 μl pipette tip when the cell confluence was about 90%. Cells was washed with PBS twice and cultured with DMEM containing 1% FBS with or without DHA for 24 h. Scratched wound was monitored, and pictures were taken at the indicated time points.

### Intracellular ROS Measurement

Esophageal cancer cell lines were seeded into a 6-well plate at a density of 5 × 10^5^ per well and treated with or without DHA or NAC for 24 h. Subsequently, DCFH-DA was diluted by 1: 1,000 with DMEM without FBS and incubated with the cells for 30 min at 37°C. After an hour, fluorescence signals in cells were observed by fluorescence microscopy, and the fluorescence intensity was measured by flow cytometry.

### RNA Extraction and Real-Time PCR

Total RNA was extracted using TRIzol reagent according to the manufacturer’s instructions. Then 1 μg of RNA was reversed transcribed into cDNA by reverse transcription system kit. Gene-specific amplification was performed in Roche Lightcycler 96 real-time PCR system (LightCycler^®^ 96 System, Roche, United States). The reaction system consisted of 5 μl 2 × TB Green Premix Ex Taq II (Takara), 0.2 μl forward primer (10 nM, Sangon, China), 0.2 μl reverse primer (10 nM, Sangon, China), 0.5 μl cDNA and 4.1 μl ddH_2_O. All measurements were performed in triplicate, the thermal cycling for indicated genes was 95°C for 30 s and followed by 35 cycles of 95°C for 5 s, 58°C for 30 s and 72°C for 20 s. The 2^−ΔΔCT^ method was used to calculate the relative expression of hTERT. Primers were used as following: hTERT forward primer: 5′-CCT​TCC​TCA​GCT​ATG​CCC​GGA​CCT-3′, hTERT reverse primer: 5′-ACA​CTT​CAG​CCG​CAA​GAC​CCC​AA-3′; β-actin forward primer: 5′-CAT​GTA​CGT​TGC​TAT​CCA​GGC-3′, β-actin reverse primer: 5′-CTCCTTAATGTCAC GCACGAT-3′.

### Western Blotting

Cells were lysed using RIPA lysis buffer containing protease inhibitors. A total of 40 µg of protein sample was used for each lane of an SDS-PAGE gel and transferred to a polyvinyldifluoride (PVDF) membrane. Afterward, the PVDF membranes were blocked with 5% skim milk for 1 h at room temperature and then probed with primary antibodies overnight at 4°C. Subsequently, PVDF membranes were incubated with secondary antibodies for 1 h at room temperature. The bands were detected by an enhanced chemiluminescence system (vilber fusion FX7, France).

### Plasmid Transfection

Esophageal cancer cell lines were seeded into 6-well plates at a density of 3 × 10^5^ per well and transfected with pcDNA3.1–3×HA-TERT or pCMV-Myc-SP1 plasmids using Lipofectamine 2000 reagent in OPTI-MEM medium after 24 h. Six hours later, medium was replaced with DMEM containing 10% FBS for 48 h.

### Tumor-Bearing Nude Mice Model Construction and Treatment

Male 4-week-old BALB/c nude mice weight around 18–20 g were purchased from Beijing Laboratory Animal Research Center (Beijing, China). Animal care and experiments protocol were approved by the Animal Ethics Committee of North Sichuan Medical College. All animals were kept in a standard environmental condition at the experimental animal center of North Sichuan Medical College (Temperature 25°C; Humidity 55%; Light/Dark cycle, 12 h light and 12 h dark). A total of 1 × 10^6^ Eca109 cells were implanted subcutaneously on the back of the nude mice (100 µl suspension). When the tumor volume of Eca109 xenografts reached about 100 mm^3^, the nude mice were randomly divided into two groups. The mice were injected intraperitoneally with DMSO or DHA every 2 days. Tumor length and width were measured with a vernier caliper, and tumor volume was calculated by the formula: Volume (mm^3^) = Length × Width^2^/2. On day 19th, the mice were sacrificed, and lung, liver, kidney, heart, and tumor tissues were collected. Tissues were fixed in 4% paraformaldehyde and embedded in paraffin for immunohistochemistry or HE stain.

### Immumohistochemical Staining

Tissue sections from paraffin-embedded esophageal cancer xenograft tissues were immunostained. Briefly, after deparaffinization and hydration of xenograft tissue sections, endogenous peroxidase was blocked by incubation with 0.3% hydrogen peroxide at 37°C for 30 min. Subsequently, sections were incubated with citrate buffer to repair antigen, and blocked with 5% BSA for 30 min at 37°C. Primary antibody was incubated at 4°C overnight and the secondary antibody was incubated at 37°C for 30 min. Finally, DAB was used as the chromogenic reagent, and counterstaining was carried out using hematoxylin. Tissue sections were dehydrated and sealed and observed with a microscope and photographed.

### HE Staining of Tissue Specimens

After deparaffinization and hydration of paraffin-embedded xenograft tissue sections, sections were stained with hematoxylin for 5 min. Then, the sections were differentiated with 1% hydrochloric acid alcohol solution, stained with eosin for 3 min. Finally, sections were dehydrated with an ethanol and xylene gradient, and were observed under microscope after being sealed with a neutral resin.

### Statistical Analysis

All *in vitro* experiments were performed three times, and data were presented as the mean ± SD. Statistical differences were evaluated by Student’s t-test or one-way analysis of variance (ANOVA) using SPSS 22.0 software. Differences with *p* < 0.05 were considered statistically significant.

## Results

### hTERT Is Highly Expressed in Esophageal Squamous Cancer and Promote Cell Proliferation and Metastasis

**FIGURE 1:**
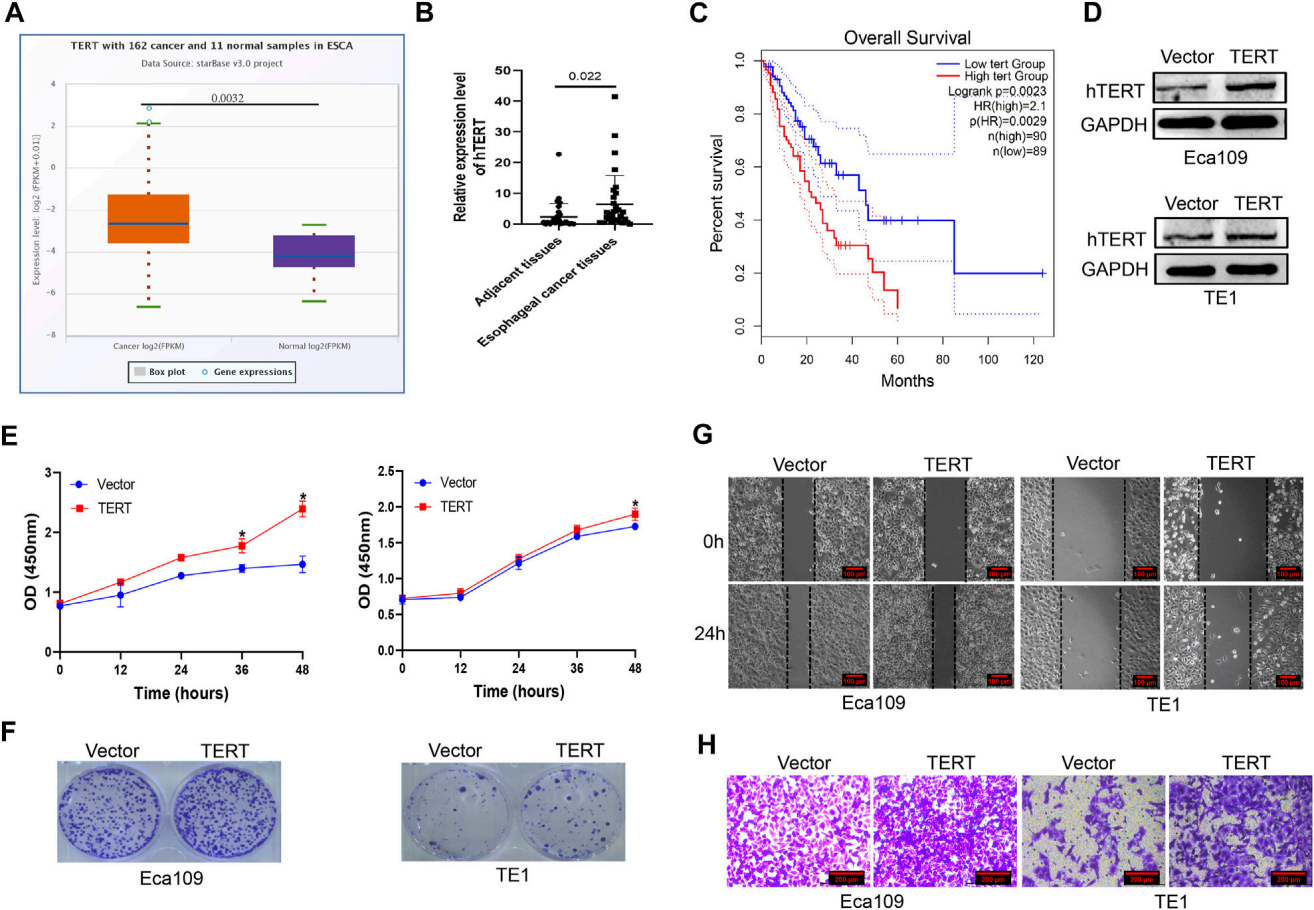
High expression of hTERT promotes the proliferation and metastasis of esophageal cancer. **(A)** The expression of hTERT mRNA in esophageal cancer from TCGA database. **(B)** The expression of hTERT mRNA in ESCC tissues and adjacent tissues (32 paired). **(C)** The correlation of hTERT with prognosis of ESCC patients from TCGA database. **(D)** The overexpression efficiency of pcDNA3.1–3×HA-TERT plasmids in Eca109 and TE1 cells. **(E)** CCK-8 assay and **(F)** plate colony formation assay were employed to determine overexpression of hTERT on the proliferation of esophageal cancer. **(G)** Scratch assay and **(H)** transwell assay were employed to determine overexpression of hTERT on the migration of esophageal cancer. (* indicate *p*< 0.05 compared with control).

Previous studies revealed that mutations in the promoter region of hTERT contributed to tumorigenesis, whether dysregulation of hTERT contribute to esophageal cancer is limited. We investigated the expression of hTERT mRNA in esophageal cancer from TCGA database and found that hTERT expression was higher in esophageal cancer than that in adjacent tissues (Figure 1A). In our cohort (*n* = 32), we found that the expression of hTERT in esophageal cancer tissues was evidently promoted, which was consistent with TCGA database (Figure 1B). Moreover, the expression of hTERT was positively correlated with worse prognosis in esophageal squamous cancer patients from TCGA database (Figure 1C). Futhermore, we attempted to uncover the association of hTERT with clinicopathological characteristics including gender, age, tumor size, tumor location, stage and lymph node metastasis, but non of these abovementioned characteristics was associated with hTERT (*p* > 0.05) (Supplementary Table S1). In order to elucidate the role of hTERT dysregulation in esophageal squamous cancer, hTERT was overexpressed in esophageal cancer cell lines Eca109 and TE1 (Figure 1D), and found that force expression of hTERT could significantly promote esophageal cancer cell proliferation and migration *in vitro* (Figures 1E–H, Supplementary Figures S1A,B). Altogether, these results demonstrated that hTERT plays an oncogenic role in esophageal squamous cancer.

### DHA Inhibites the Proliferation and Metastasis of Esophageal Squamous Cancer Cells and Downregulated the Expression of hTERT

**FIGURE 2:**
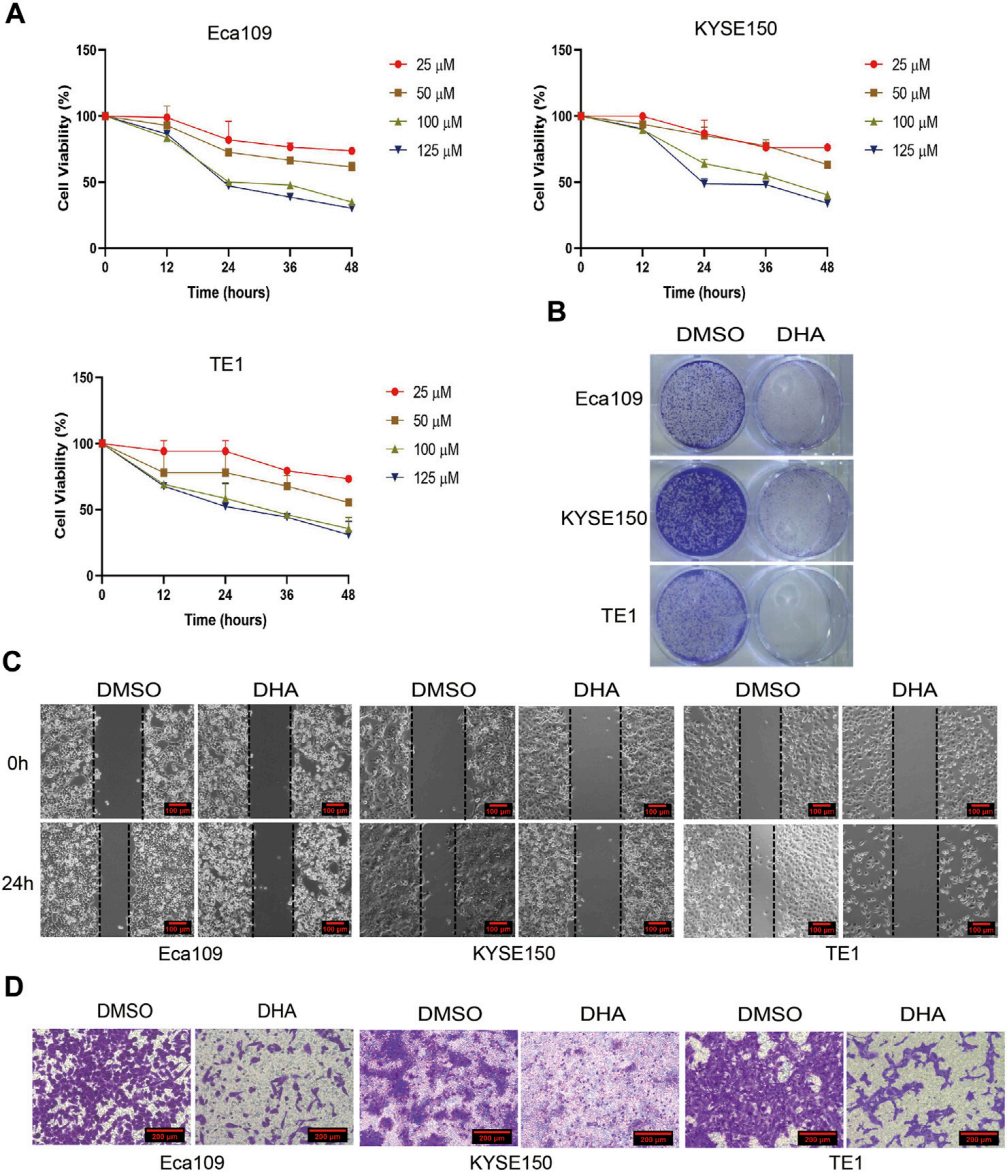
DHA inhibits the proliferation and migration of esophageal cancer. **(A)** Cell viability of Eca109, KYSE150, and TE1 was detected by CCK8 assay after treated with indicated concentrations of DHA (0,25, 50, 100,125 μM). **(B)** Represent images of plate colony formation assay from esophageal cancer cell lines Eca109, KYSE150, and TE1 treated with DHA (100 μM) or DMSO. **(C)** Represent images of scratch assay and **(D)** transwell assay from esophageal cancer cell lines Eca109, KYSE150, and TE1 treated with DHA (100 μM) or DMSO.

Our previous study showed that DHA induced cell cycle arrest in Eca109 cells *in vitro* and *in vivo* (Ma et al., 2020). Whether the inhibition effect is universal or if other mechanisms involve in DHA treated esophageal cancer cell lines is unknown. Hence, we investigated the effect of DHA on the proliferation and metastasis of esophageal cancer cells Eca109, KYSE150, and TE1. As shown in Figure 2A, DHA significantly inhibited the viability of esophageal cancer cell lines (Eca109, KYSE150, and TE1) in a dose- and time-dependent manner. Plate colony formation assay showed that 100 μM DHA evidently inhibited the proliferation of esophageal cancer cells (Figure 2B). Moreover, transwell assay and scratch experiment revealed that DHA significantly inhibited esophageal cancer cells migration compared with cells treated with DMSO (Figures 2C,D, Supplementary Figures S1C,D). Taken together, the abovementioned results showed that DHA significantly inhibit the proliferation and migration of esophageal cancer cells.

**FIGURE 3:**
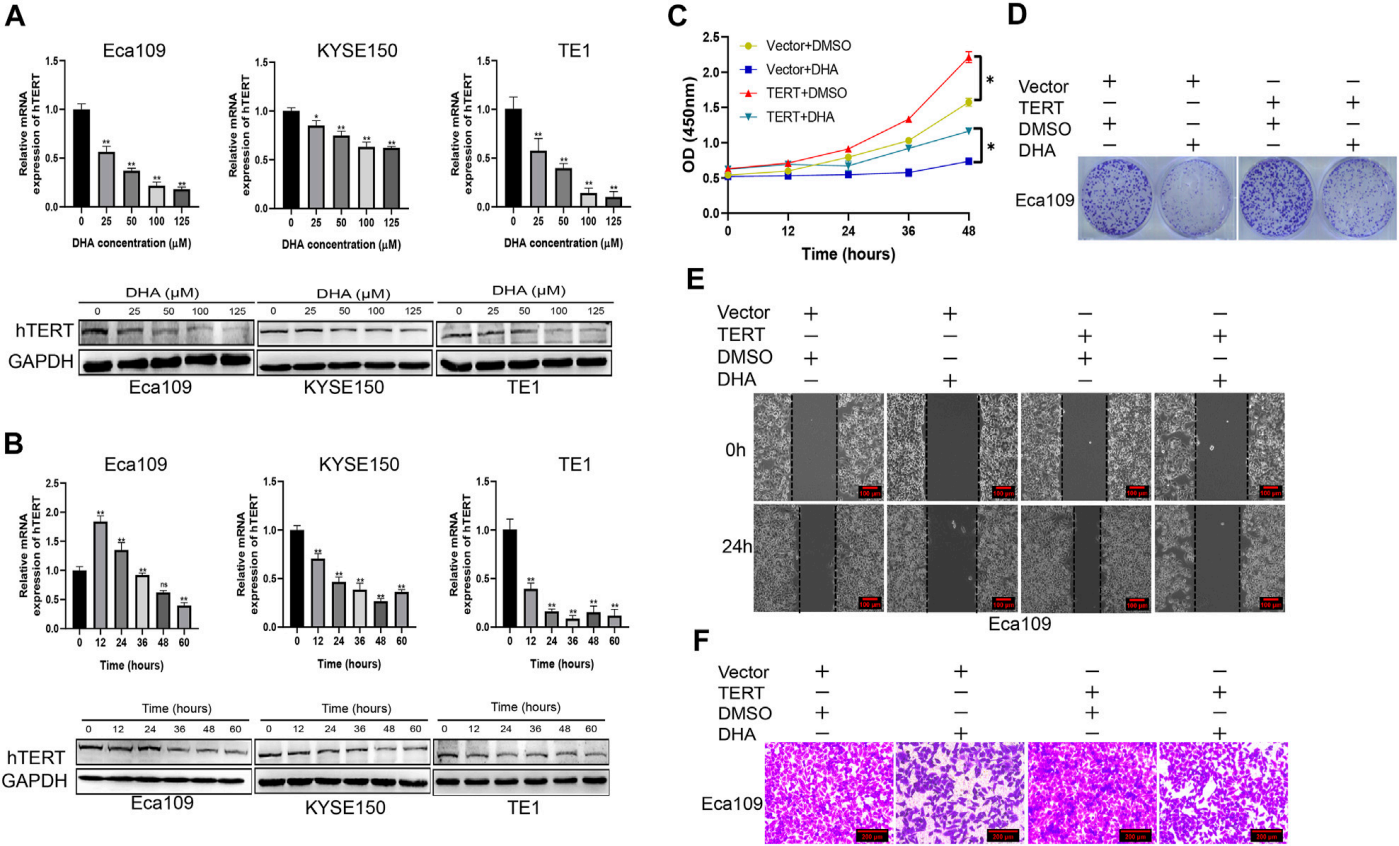
DHA inhibits the proliferation and migration of esophageal cancer through downregulating the expression of hTERT. **(A)** The expression of hTERT on mRNA (upper) and protein (lower) levels in Eca09, KYSE150, and TE1 cells after treated with indicated concentration of DHA for 48 h. **(B)** The expression of hTERT on mRNA (upper) and protein (lower) levels in Eca09, KYSE150, and TE1 cells after treated with DHA (100 μM) at indicated time points. **(C,D)** The effect of overexpression of hTERT on the proliferation of Eca109 cells treated with DHA and evaluated by the CCK8 assay **(C)** and colony formation assay **(D)**. **(E,F)** Scratch assay **(E)** and transwell assay **(F)** showing *in vitro* migration of Eca109 cells overexpressing hTERT and treated with DHA. (* indicate *p*< 0.05 and ** indicate *p*< 0.01 compared with control).

Cause hTERT plays an oncogenic role in esophageal squamous cancer, we wonder whether DHA regulates the expression of hTERT in esophageal cancer cell lines. We measured the expression of telomerase reverse transcriptase (hTERT) on mRNA and protein levels in Eca109, KYSE150, and TE1 cells treated with indicated concentrations of DHA. The results showed that DHA markedly downregulated the expression of hTERT on mRNA and protein levels in esophageal cancer cells in a dose-dependent manner (Figure 3A). Moreover, 100 μM DHA also inhibited the expression of hTERT in a time-dependent manner (Figure 3B). Furthermore, we elucidated whether overexpression of hTERT could attenuate the anticancer activity of DHA. We found that overexpression of hTERT in esophageal cancer cells could alleviate the inhibition of DHA on the proliferation and migration of Eca109 cells (Figures 3C–F, Supplementary Figure S1E). These results demonstrated that hTERT was an oncogene in esophageal squamous cancer and could be used as a therapeutic target of DHA.

### DHA Downregulates the Expression of hTERT in Esophageal Cancer Through Inhibiting Transcriptional Factor SP1

**FIGURE 4:**
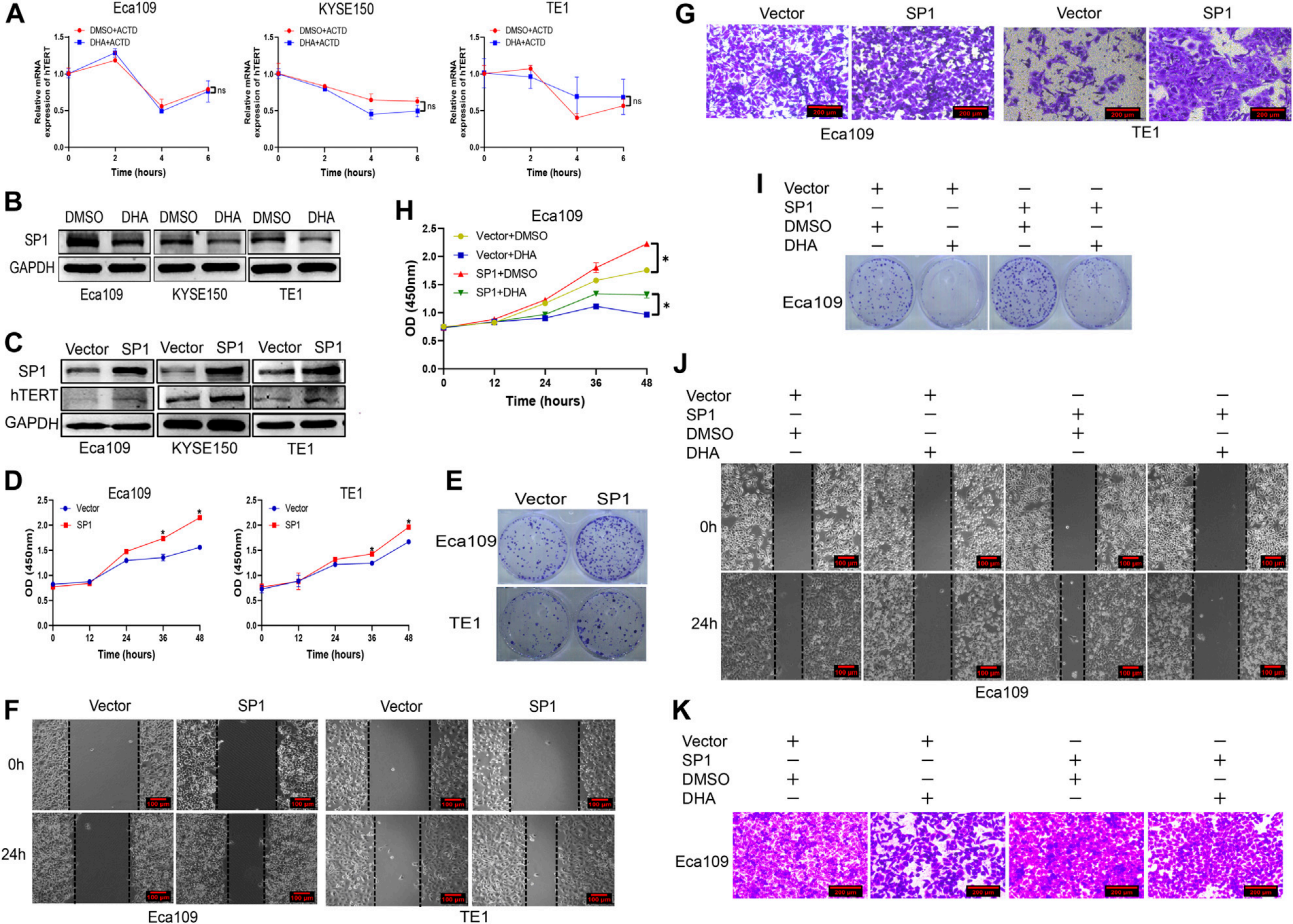
DHA downregulates the expression of hTERT in esophageal cancer through inhibiting transcriptional factor SP1. **(A)** The effect of DHA on hTERT mRNA stability in Eca109, KYSE150, and TE1 cells evaluated by actinomycin D assays. **(B)** DHA downregulates the expression of transcription factor SP1 in Eca109, KYSE150, and TE1 cells. **(C)** Overexpression of SP1 enhances the expression of hTERT. **(D,E)** Overexpression of SP1 promoted the proliferation of Eca109 and TE1 cells determined by the CCK8 **(D)** and plate colony formation assays **(E)**. **(F,G)** Overexpression of SP1 promoted the migration of Eca109 and TE1 cells determined by the scratch **(F)** and transwell assays **(G)**. **(H,I)** The proliferation of SP1-overexpressing Eca109 cells after treated with DHA and measured by the CCK8 **(H)** and colony formation assays **(I)**. **(J,K)** The migration of SP1-overexpressing Eca109 cells after treatment with DHA and evaluated by scratch assay **(J)** and transwell assay **(K)**. (* indicate *p*< 0.05 compared with control).

We further explored the molecular mechanism by which DHA downregulates hTERT expression in esophageal cancer cells. It is well-known that regulation of gene expression is mainly at the transcriptional and post-transcriptional levels. However, it is unclear whether DHA regulates hTERT expression at the transcriptional or post-transcriptional level. We firstly measured the mRNA stability of hTERT in DHA treated esophageal cancer cells by using actinomycin D experiments, and found that the stability of hTERT mRNA was not significantly difference between cells treated with DHA and vehicle (Figure 4A). Therefore, we speculated that downregulation of hTERT by DHA might trough the post-transcriptional level. It has been reported that the core promoter region (−181 bp) of hTERT contains two c-Myc and SP1 binding sites, and c-Myc and SP1 have been verified to upregulate telomerase activity through promoting the transcription of hTERT (Kyo et al., 2000). Since c-Myc and SP1 are directly involved in the expression of hTERT, we further determined whether DHA regulates c-Myc and SP1 protein expression in esophageal cancer cells. As shown in Figure 4B, DHA could downregulate the expression of SP1, but not c-Myc (data not shown), in Eca109, KYSE150, and TE1 cells. Moreover, overexpression of SP1 in esophageal cancer cells significantly upregulated the expression of hTERT (Figure 4C). In addition, we found that overexpression of SP1 enhanced the proliferation (Figures 4D,E) and migration (Figures 4F,G, Supplementary Figures S1F,G) ability of Eca109 and TE1 cells. Meanwhile, overexpression SP1 could attenuate the inhibition effect of DHA on the proliferation and migration ability in Eca109 cells (Figures 4H–K, Supplementary Figures S1H,I). Altogether, these data indicated that DHA downregulated hTERT expression through inhibiting transcriptional factor SP1 in esophageal cancer cells, subsequently inhibiting the esophageal cancer cells proliferation and migration.

### ROS Plays a Vital Role in DHA Induced hTERT Downregulation in Esophageal Cancer

**FIGURE 5:**
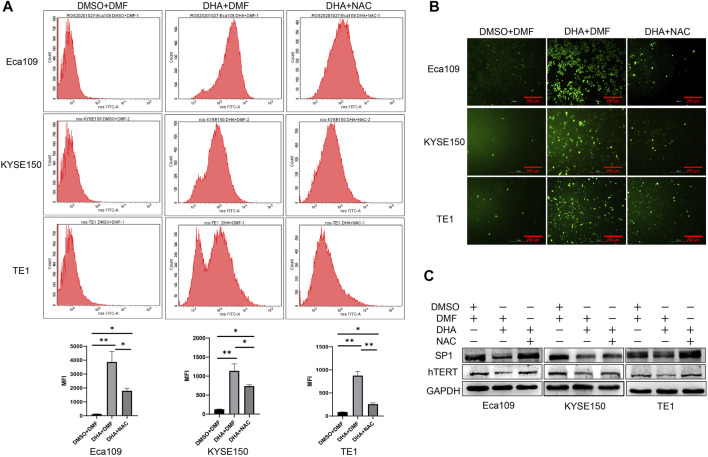
DHA induces intracellular ROS generation in Eca109, KYSE150, and TE1 cells after treated with DHA. **(A)** The mean fluorescence intensity (MFI) of cells treated with or without DHA or NAC and the statistical charts. **(B)** Images from a fluorescence microscope after cells treated with or without DHA or NAC followed by DCFH-DA co-incubation. **(C)** The expression of SP1 and hTERT protein in cells treated with or without DHA or NAC. (* indicate *p*< 0.05 and ** indicate *p*< 0.01 compared with control).

Some studies have reported that DHA exerted antitumor effects mainly through the induction of ROS production (Xu et al., 2016). Camptothecin (CPT) can promote hTERT expression and telomerase activity by inducing ROS production, leading to SP1 activation (Dilshara et al., 2019). In RD and Rh30 rhabdomyosarcoma (RMS) cells, Methyl 2-trifluoromethyl-3,11-dioxo-18β-olean-1,12-dien-3-oate (CF3DODA-Me)can downregulate the expression of SP1 by inducing ROS (Kasiappan et al., 2019). Our previous study also revealed that DHA induced ROS production in Eca109 cells (Ma et al., 2020), whether DHA can induce ROS in other esophageal cancer cells is unknown. Therefore, we determined ROS level in DHA treated esophageal cancer cell lines Eca109, KYSE150, and TE1 by fluorescence microscopy and flow cytometry. As shown in Figures 5A,B, DHA significantly induced intracellular ROS production in esophageal cancer cells, and the antioxidant NAC (N-acetyl-l-cysteine) could alleviate DHA induced ROS production. In addition, Western Blot results indicated that NAC could attenuate the downregulation of SP1 and hTERT protein expression caused by DHA (Figure 5C). These results verified that DHA inhibits hTERT transcription by inducing ROS production and down-regulating transcriptional factor SP1.

### DHA Inhibits Tumor Growth in Esophageal Cancer Xenograft Mouse Models

**FIGURE 6:**
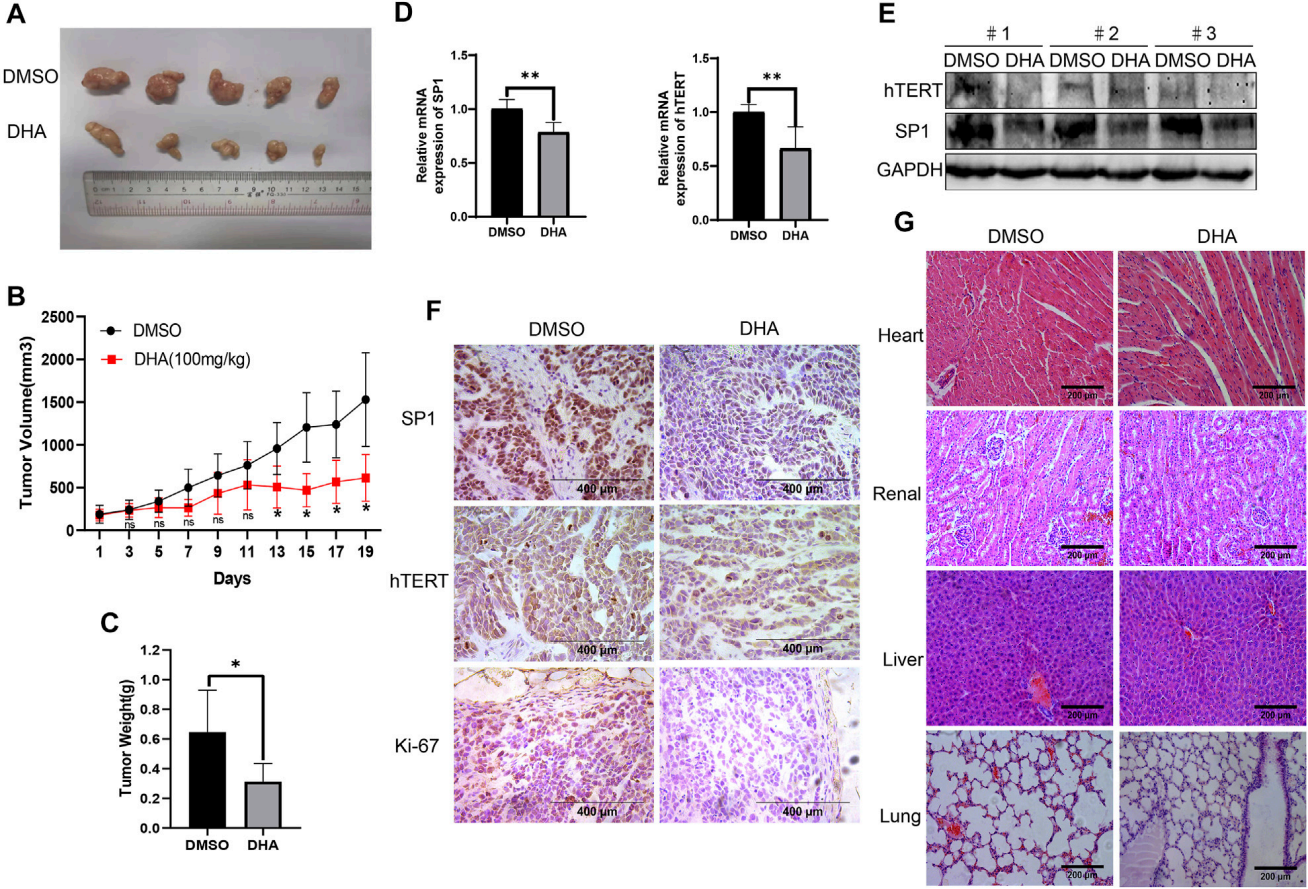
DHA suppresses tumor growth of ESCC *in vivo*. **(A)** After mice were sacrificed on day 19th, the tumors were stripped and photographed. **(B,C)** The proliferation curve **(B)** and tumor weight **(C)** of tumors in xenograft mouse model treated with DHA or vehicle. **(D,E)** The expression of SP1 and hTERT in tumor tissues detected by PCR and Western blot. **(F)** Immunohistochemical staining for SP1, hTERT, and Ki-67 of tumor tissues from xenograft mouse model. **(G)** HE staining of heart, liver, lung, and kidney in xenograft mouse model. (* indicate *p*< 0.05 and ** indicate *p*< 0.01 compared with control).

To investigate the anti-esophageal cancer effect of DHA *in vivo*, we established Eca109 tumor-bearing nude mouse models. As shown in Figure 6A,B, the tumor sizes were significantly decreased in the DHA-treated group compared to the DMSO-treated group. Moreover, the tumor weight was lower (Figure 6C) in DHA treated Eca109 xenograft mouse models. Furthermore, DHA evidently downregulated the expression of SP1 and hTERT on mRNA and protein level in tumor tissues (Figures 6D,E). The IHC results also elucidated that the expression of hTERT and SP1 significantly downregulated in DHA treated xenograft esophageal cancer tissues, as well as the proliferation marker Ki-67 (Figure 6F). Intriguingly, side effect of DHA on heart, liver, lung, and kidney tissues of xenograft mouse models was evaluated by HE staining, and the results revealed that DHA exhibit no significant toxicity on these key organs of the mouse model (Figure 6G). Taken together, these data suggested that DHA can inhibit the growth of esophageal cancer *in vivo* without significant toxicity.

## Discussion

**FIGURE 7:**
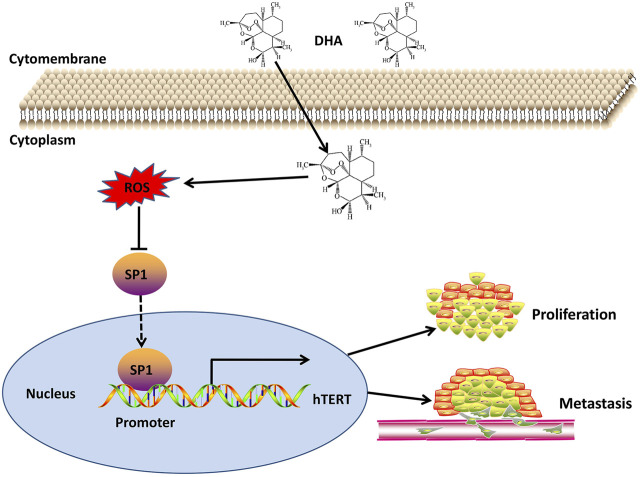
Schematic diagram of the mechanism of DHA against esophageal cancer through ROS/SP1/hTERT axis.

In this study, we investigated the expression of hTERT in esophageal cancer by TCGA database, and subsequently investigated the anticancer effect of DHA on esophageal cancer cells by *in vitro* and *in vivo* experiments. We for the first time demonstrated that DHA inhibited the proliferation and metastasis of esophageal cells through ROS-SP1-hTERT axis (Figure 7). Moreover, overexpression of SP1 or hTERT enhanced the proliferation and migration of esophageal cancer cells, and attenuated the inhibitory effect of DHA on the proliferation and migration of esophageal cancer cells. These findings revealed the anticancer effects of DHA and its positive feedback regulatory pathway in esophageal cancer cells.

Human telomerase reverse transcriptase (hTERT) is a catalytic subunit of telomerase that has been shown to regulate telomerase activity and play a key role in tumorigenesis and proliferation of cancer cells (Pal et al., 2013). It has been reported that over 85–90% of cancer cells are found to upregulate telomerase expression, conferring them the potential for unlimited proliferation (Ramlee et al., 2016). Studies have shown that hTERT promoter mutation and epigenetic activation are the main factors of abnormal telomerase activity (Barthel et al., 2017). At present, most of the studies focus on the mutations of hTERT promoter region. Recurrent hTERT promoter mutations were first reported in a family of patients with melanoma, and these mutations increase the transcriptional activity of the hTERT promoter (Horn et al., 2013; Huang et al., 2013). Subsequently, researchers found that hTERT promoter mutations were also present in liposarcoma, hepatocellular carcinoma, urothelial carcinoma, tongue scale cell carcinoma, medulloblastoma, and glioma, and were associated with telomerase activity (Killela et al., 2013). In our study, using TCGA database, we found that hTERT mRNA was highly expressed in esophageal cancer and positively correlated with worse prognosis. In our cohort, the expression of hTERT mRNA in esophageal cancer tissues (*n* = 32) was evidently upregulated, which was consistent with the results from the TCGA database. However, we failed to find the correlation between hTERT mRNA expression and clinicopathological characteristics, which might be due to the limited sample size. Therapeutics targeting telomerase and hTERT currently have been developed (Sprouse et al., 2012). The most common therapeutic methods targeting hTERT including oligonucleotide inhibitors, small-molecule telomerase inhibitors, immunotherapeutic approaches, telomerase-directed gene therapy, and phytochemicals (Jager and Walter, 2016). The oligonucleotide imetelstat (GRN163L) is the only anti-telomerase compound widely evaluated in clinical trials, which targets the RNA template of hTERT by binding to the catalytic site of telomerase (Harley, 2008). Telomerase-based cancer immunotherapy has two strategies, including the hTERT vaccine approach and the dendritic cell approach. Clinical trials of three hTERT vaccines such as GV1001, Vx001, and GRNVAC1 have shown that they can stimulate CD4^+^ and CD8^+^ T cells responses in telomerase-positive tumors with minimal effect on normal cells (Jafri et al., 2016). The small molecule telomerase inhibitor BIBR1532 inhibits telomerase catalytic subunit (hTERT) binding (Arndt & MacKenzie, 2016). In addition, a variety of naturally occurring compounds or phytochemicals in plants are also proved to inhibit telomerase activity in various cancers, such as boldine and gambogicacid (Chen and Zhang, 2016).

Artemisinin is also a kind of phytochemical with effective antimalarial effect, which extracted from Traditional Chinese Medicine artemisia annua. DHA is an active metabolite of artemisinin compounds and has stronger antimalarial activity than artemisinin. Compared with artemisinin, DHA is also considered to have the advantages of high efficiency and low toxicity (Efferth et al., 2001; Karunajeewa et al., 2007). In the present study, no apparent pathological changes were found in heart, liver, lung, and kidney of the xenograft model between the DHA-treated group and the control group. These results indicated that DHA was a safe potential antitumor reagent with low toxicity. Besides the antimalaria activity, DHA has been reported to exhibit significant anticancer activity in a variety of tumors (Dai et al., 2021), including lung cancer (Jiang et al., 2016), ovarian cancer (Li et al., 2018), MM (Wu et al., 2020), and hepatocellular carcinoma (Zou et al., 2019). Previous study demonstrated that inhibition of telomerase activity could reduce the proliferation and migration of esophageal cancer cells (Li et al., 2020). Our data showed that DHA could evidently downregulate the expression of hTERT both on mRNA and protein level. We then used actinomycin D experiments to evaluate the stability of hTERT in esophageal cancer cells after treated with DHA, and found that DHA did not affect hTERT mRNA stability, which suggested that DHA down-regulating hTERT expression might be at the post-transcriptional level. Studies have reported that the activity of TERT promoter is usually regulated by a variety of transcription factors, such as c-Myc, NF-κB, SP1, and STAT3 (Ramlee et al., 2016). We evaluated the expression of the abovementioned transcriptional factors in DHA treated Eca109, KYSE150, and TE1 cells, and found that only SP1 was downregulated by DHA. Meanwhile, overexpression of SP1 in Eca109 and TE1 cells obviously promoted hTERT protein expression and enhanced esophageal cells proliferation and migration. This result suggested that SP1 was a transcriptional activator of hTERT in esophageal cancer cells. This was similar to Kuhlmann’s reported that in adult T-cell leukemia (ATL) patients, the human T-cell leukemia virus type 1 protein HTLV-1bZIP factor (HBZ) leaded to target gene activation by forming a heterodimer with Jund and interacting with SP1 in the hTERT promoter region (Kuhlmann et al., 2007). Another study reported that MBD1 containing chromatin-associated factor 1 (MCAF1) in HeLa cells interacted with SP1 and general transcriptional machinery to promote hTERT expression (Liu et al., 2009). Moreover, we constructed a xenograft model and found that SP1 and hTERT mRNA and protein levels were significantly downregulated in the DHA treatment group compared with the DMSO group. Furthermore, overexpression of SP1 or hTERT could attenuate the inhibition of DHA on the proliferation and migration of esophageal cancer. This phenomenon indicates that both SP1 and hTERT genes are essential in esophageal cancer and are involved in the proliferation and metastasis of esophageal cancer cells.

The anti-tumor and antimalarial mechanism of DHA depended on the presence of ferrous ions, which breaks the peroxide bridge in DHA and leads to the production of cytotoxic reactive oxygen species (ROS) (Efferth, 2017). In our study, we found that DHA significantly induced intracellular ROS production in Eca109, KYSE150, and TE1 cells, and the antioxidant NAC attenuated the ROS production in Eca109, KYSE150, and TE1 cells induced by DHA. It has been shown that in JEG-3 cells, ROS up-regulated the expression level of miR-335–5p in a p53-dependent manner, resulting in downregulation of SP1 expression (Lu et al., 2020). In the present study, we found that inhibition of ROS by NAC significantly reverse DHA induced downregulation of SP1 and hTERT. These results demonstrated that SP1 and hTERT downregulation in DHA treated esophageal cancer cells was ROS-dependent.

In the current study, we demonstrated that DHA inhibited the proliferation and metastasis of esophageal cells through ROS-SP1-hTERT axis. However, we failed to reveal the effect of DHA induced ROS on the proliferation and migration ability of esophageal cancer cells. In summary, we provide evidence of a novel anticancer mechanism of DHA in esophageal cancer and provide useful evidence for the potential clinical application of DHA.

## Data Availability

The original contributions presented in the study are included in the article/Supplementary Material, further inquiries can be directed to the corresponding authors.

## Supplementary Material

Supplementary Figure S1 The quantitative results of scratch assay and transwell assay. **(A,B)** The quantitative results of scratch assay from Figure 1G **(A)** and quantitative results of transwell assay from Figure 1H **(B)**. **(C,D)** The quantitative results of scratch assay from Figure 2C **(C)** and quantitative results of transwell assay from Figure 2D **(D)**. **(E)** The quantitative results of scratch assay and transwell assay from Figures 3E,F. **(F,G)** The quantitative results of scratch assay from Figure 4F **(F)** and quantitative results of transwell assay from Figure 4G **(G)**. **(H,I)** The quantitative results of scratch assay from Figure 4J **(H)** and quantitative results of transwell assay from Figure 4K **(I)**. (* indicate *p*< 0.05, ** indicate *p*< 0.01, and ***indicate *p*< 0.001 compared with control.)

Supplementary Table S1 Clinical parameters of 32 ESCC patients association with hTERT expression levels.
