# Supplementary figures and images for: Human Telomerase Reverse Transcriptase as a Therapeutic Target of Dihydroartemisinin for Esophageal Squamous Cancer
Source: Front Pharmacol. 2021 Oct 22;12:769787. doi: 10.3389/fphar.2021.769787 (PMC8569230; doi:10.3389/fphar.2021.769787)

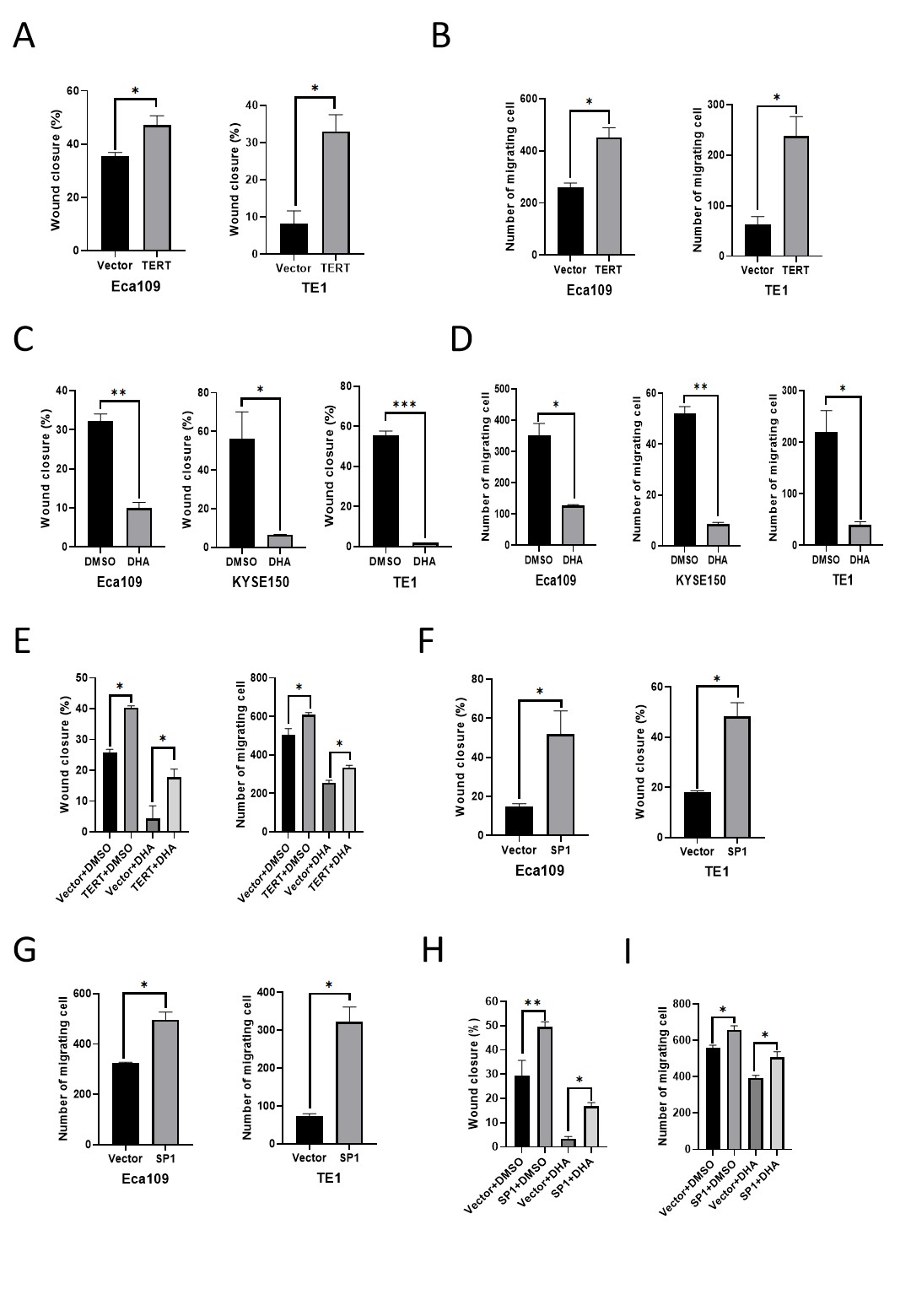

Supplement: Supplementary file 1 [file Image1.JPEG]
