# Supplementary material for: Human Telomerase Reverse Transcriptase as a Therapeutic Target of Dihydroartemisinin for Esophageal Squamous Cancer
Source: Front Pharmacol. 2021 Oct 22;12:769787. doi: 10.3389/fphar.2021.769787 (PMC8569230; doi:10.3389/fphar.2021.769787)
Supplement: Supplementary file 2 [file Table1.DOC]

Table 1. Clinical parameters of 32 ESCC patients association with hTERT expression levels.

| **Variable** | **hTERT expression** | | **X2** | **P** |
| --- | --- | --- | --- | --- |
|  | **High（n=24） Low (n=8)** | |  |  |
| **Age(years)** |  |  |  | **0.423** |
| **＜65** | **10** | **5** |  |  |
| **≥65** | **14** | **3** |  |  |
| **Sex** |  |  |  | **0.386** |
| **Male** | **16** | **7** |  |  |
| **Female** | **8** | **1** |  |  |
| **Tumor Location** |  |  | **1.556** | **0.459** |
| **Up** | **2** | **2** |  |  |
| **Middle** | **19** | **5** |  |  |
| **Down** | **3** | **1** |  |  |
| **Differentiation** |  |  | **1.876** | **0.393** |
| **High** | **6** | **4** |  |  |
| **Middle** | **15** | **3** |  |  |
| **Low** | **3** | **1** |  |  |
| **Tumor size (cm)** |  |  | **0.985** | **0.611** |
| **＜3** | **3** | **2** |  |  |
| **3-5** | **20** | **6** |  |  |
| **＞5** | **1** | **0** |  |  |
| **Lymph node metastasis** |  |  |  | **0.116** |
| **Positive** | **7** | **5** |  |  |
| **Negative** | **17** | **3** |  |  |
| **Tumor Staging** |  |  |  | **0.807** |
| **Ⅰ-Ⅱ** | **19** | **6** |  |  |
| **Ⅲ-Ⅳ** | **5** | **2** |  |  |

*X*^2^ Test was used to examine the association of categorical variables.
**P* < 0.05, ***P* < 0.01.
